# The use of negative-pressure wound therapy after total knee arthroplasty is effective for reducing complications and the need for reintervention

**DOI:** 10.1186/s12891-020-03510-z

**Published:** 2020-07-25

**Authors:** Camilo Partezani Helito, Marcel Faraco Sobrado, Pedro Nogueira Giglio, Marcelo Batista Bonadio, José Ricardo Pécora, Marco Kawamura Demange, Riccardo Gomes Gobbi

**Affiliations:** 1 Grupo de Joelho, Instituto de Ortopedia e Traumatologia, Hospital das Clínicas HCFMUSP, Faculdade de Medicina, Universidade de São Paulo, Rua Dr. Ovídio Pires de Campos, 333, Cerqueira Cesar, São Paulo, SP – CEP: 05403-010 Brazil; 2 Hospital Sírio Libanês, São Paulo, Brazil

**Keywords:** Negative-pressure wound, Wound dehiscence, Knee arthroplasty, Wound complication, Periprosthetic joint infection

## Abstract

**Background:**

Wound healing complications are causal factors of prosthesis infection and poor postoperative evolution of patients after total knee arthroplasty (TKA). Negative-pressure wound therapy (NPWT) can be an option to minimize these complications. The aim of this study is to compare the complications of patients undergoing TKA who used a portable NPWT device in the immediate postoperative period with those of a control group.

**Methods:**

A total of 296 patients were evaluated. Patients were divided into two groups: those who used NPWT for seven days in the postoperative period (Group 1 – prospective evaluated) and those who used conventional dressings (Group 2 – historical control group). Epidemiological data, comorbidities, local parameters related to the surgical wound and complications were evaluated.

**Results:**

The groups did not differ in regard to sex, age and clinical comorbidities. Overall, 153 (51.7%) patients had at least one risk factor for wound complications. Patients who used NPWT had a lower rate of complications (28.5% vs. 45.7%, *p* = 0.001) and a lower rate of reintervention in the operating room (2% vs. 8.5%, p = 0.001). Patients in group 1 had a lower incidence of hyperaemia (14.7% vs. 40.2%, *p* = 0.01), skin necrosis (2.1% vs. 8.5%, *p* = 0.04) and wound dehiscence (3.1% vs 10.1%, *p* = 0.03). The use of NPWT was a protective factor for the presence of complications, with an odds ratio of 0.36 (95% CI 0.206–0.629).

**Conclusion:**

The number of complications related to the wound after TKA is high; however, most of them are minor and have no impact on the treatment and clinical evolution of patients. The use of NPWT decreased the number of surgical wound complications, especially hyperaemia, dehiscence and necrosis, and reduced the need for reintervention.

## Background

The number of patients who undergo total knee arthroplasty (TKA) has been increasing in recent years and is expected to grow exponentially in the coming decades [1]. One of the most feared and difficult to treat complications is periprosthetic infection, which occurs in approximately 1 to 2% of cases, according to recent studies [2, 3]. One of the causal factors of prosthesis infection and the poor postoperative evolution of patients is complications related to wound healing [4, 5]. Adelani et al. showed that patients re-admitted to the hospital after arthroplasty due to non-infectious wound complications had worst knee function and more pain [6]. In addition, surgical wound complications, such as dehiscence, persistent drainage, and haematomas, may predispose patients to infection [7, 8].

To minimize the complications related to wound healing after knee arthroplasty, several pre-, intra- and postoperative factors can be addressed, such as the control of clinical comorbidities before the surgical procedure, the use of less traumatic surgical techniques, careful haemostasis of the operative field and wound closure without tension, in addition to early control of eventual persistent drainage or of poor evolution of the wound in the early postoperative period [5]. Even with all the necessary precautions and care, some patients do not progress satisfactorily, especially those with an increased risk of postoperative wound healing complications, such as patient who are obese, smoke or have diabetes and patients with systemic inflammatory diseases, such as rheumatoid arthritis [9–13].

One of the recent alternatives that has been used to improve wound healing after TKA and to minimize wound complications and the incidence of infection is the use of negative-pressure therapy [14, 15]. This type of therapy has been used successfully in several fields of medicine, such as plastic surgery, colorectal surgery and caesarean, and has achieved excellent results in the orthopaedic field, especially in cases of open fractures or open wounds [16–18]. In closed wounds, as is the case of postoperative use after arthroplasties, its benefit is still controversial, with a lack of agreement among existing studies regarding its actual benefit [19, 20]. Most studies, however, only evaluated major complications, such as infection or need for reintervention due to dehiscence of or persistent drainage from the surgical wound, and did not include in the analyses any minor wound complications that can also delay rehabilitation and worsen functional outcomes.

Therefore, the aim of the present study was to compare major and minor complications in patients who underwent TKA and used portable negative-pressure wound therapy (NPWT) in the immediate postoperative period with those in patients who used conventional dressings (control group). Our hypothesis is that negative-pressure therapy will reduce wound healing complications and the rate of periprosthetic infections.

## Methods

**Fig. 1:**
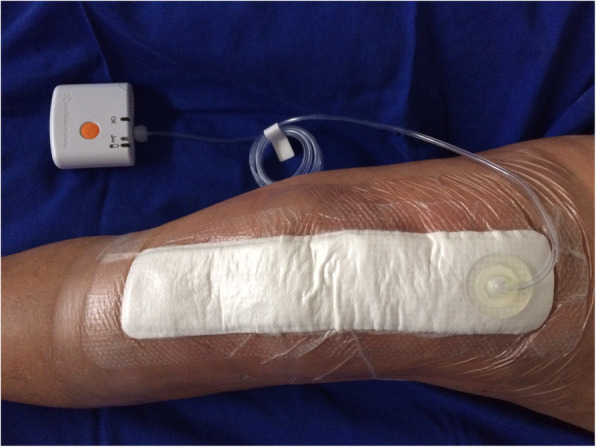
Patient with a left total knee arthroplasty using the PICO® device for negative-pressure wound therapy in the immediate postoperative period

A consecutive non-randomized case series of patients undergoing TKA who used portable NPWT (PICO®, Smith Nephew) immediately after wound closure in the operating room and for 7 consecutive days were prospectively evaluated from January 2016 to December 2017 (Fig. 1). The inclusion criteria were patients of any age with primary or secondary knee osteoarthritis who underwent elective unilateral arthroplasty. The exclusion criteria were patients with previous knee, femur or tibia surgeries on the side of the arthroplasty, previous osteomyelitis in the femur or tibia ipsilateral to the operated knee and patients who required the use of revision implants in the tibia or femur due to severe deformity or previous severe ligament instability. In addition, patients who were unable to perform the weekly postoperative evaluation in person in accordance with the established schedule were excluded.

Surgery was performed with the use of a pneumatic tourniquet through an anterior knee incision and trans quadricipital and medial parapatellar approach. The procedures were always performed with resection of the posterior cruciate ligament (PCL). All cases were performed with cemented components without antibiotics. The subcutaneous tissue was closed with 3–0 Vicryl sutures, and the skin was closed with simple 4–0 nylon sutures. Antibiotic prophylaxis was performed with cefuroxime for 24 h for all cases. Drains were used for a maximum period of 24 h, regardless of the draining volume, and the sutures were removed between the 14th and 21st days after surgery, according to the outpatient return date. Post-operative rehabilitation was not changed due the NPWT device, as its application does not hinder patient’s functional exercises. Range of motion exercises and weight-bearing as tolerated were allowed since the first day after surgery. Anticoagulant medication was used for venous thromboembolism prophylaxis for 14 days. Pain control was performed with conventional analgesics and opioid medication if needed.

This group was compared with a control group of consecutive cases (surgeries performed from January 2013 to December 2015) that underwent the same type of intervention using the same technique but who used conventional dressings; the first dressing was changed after 7 days, except in cases of saturation due to bleeding from the wound.

All surgical wound healing complications were evaluated weekly in the first 6 weeks. After that, the cases that progressed satisfactorily were evaluated at 3 months, 6 months and 12 months to assess eventual infections. The cases that progressed with problems or complications related to the surgical wound were evaluated weekly until resolution of the condition. The patients who used the PICO® device were evaluated when it stopped functioning after 7 days, unless there was a prior need due to saturation of the dressing.

**Fig. 2:**
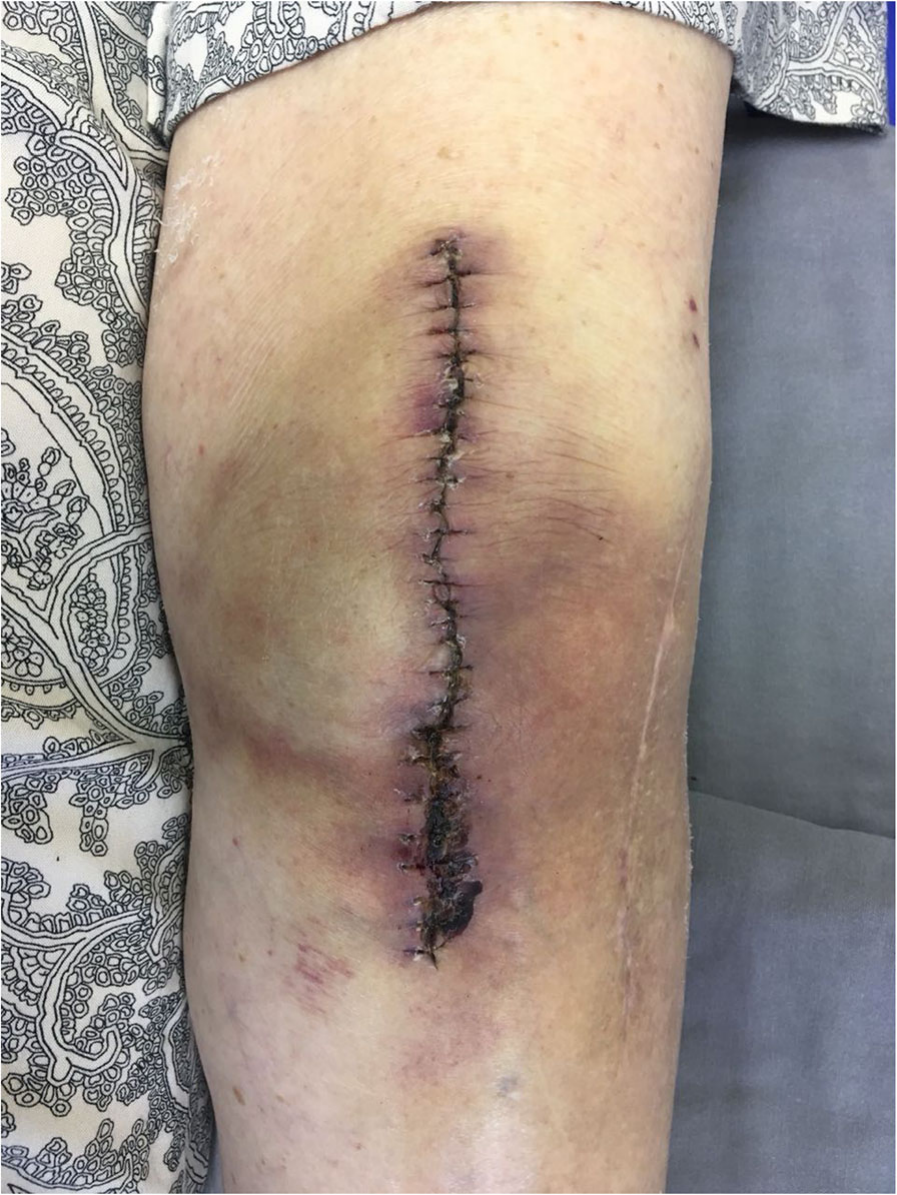
Edge necrosis in the lateral and distal portion of the surgical wound and hyperaemia around the wound of a patient (smoker) who underwent total primary arthroplasty of the left knee and used conventional dressings; 14 days postoperatively

**Fig. 3:**
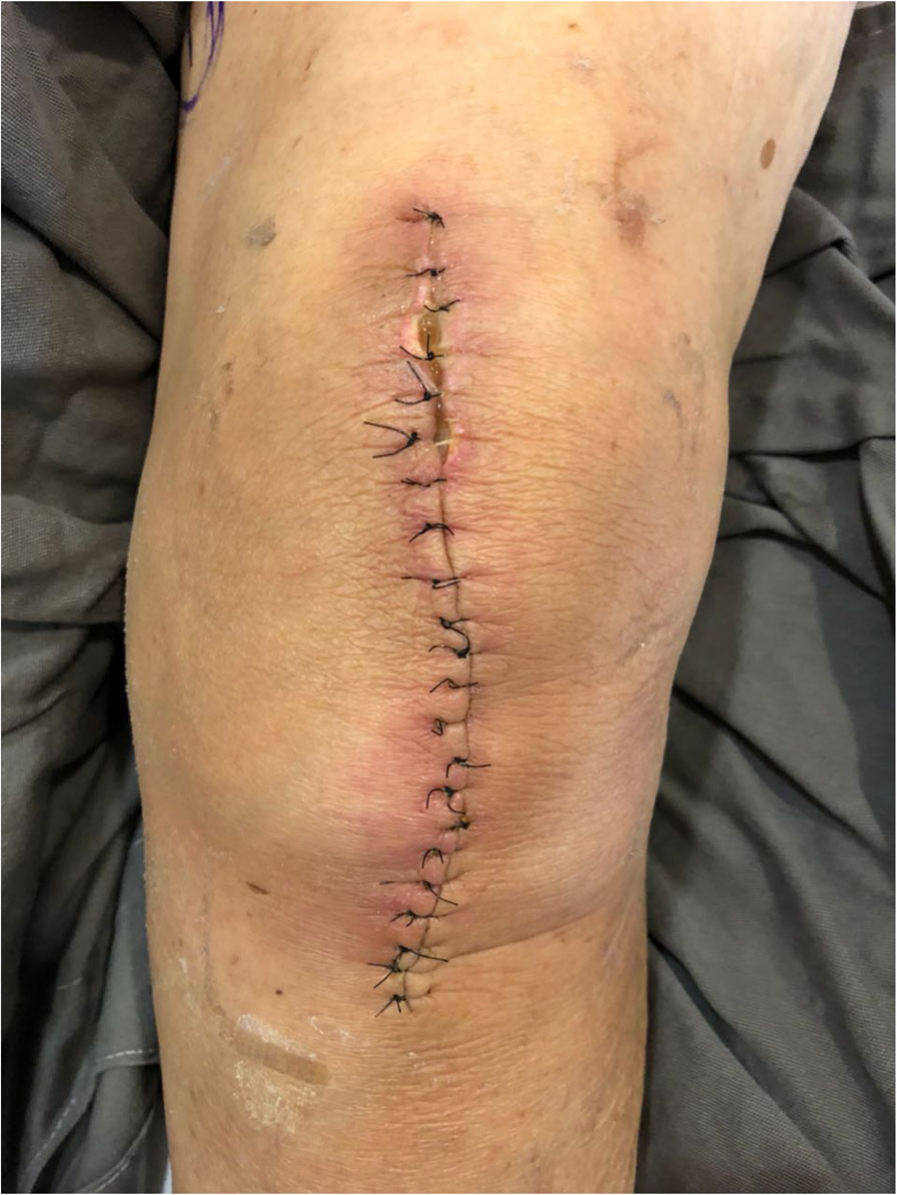
Proximal dehiscence of the surgical wound and hyperaemia around the entire wound of a patient without clinical comorbidities who underwent primary total arthroplasty of the left knee and used conventional dressings; 14 days postoperatively

**Fig. 4:**
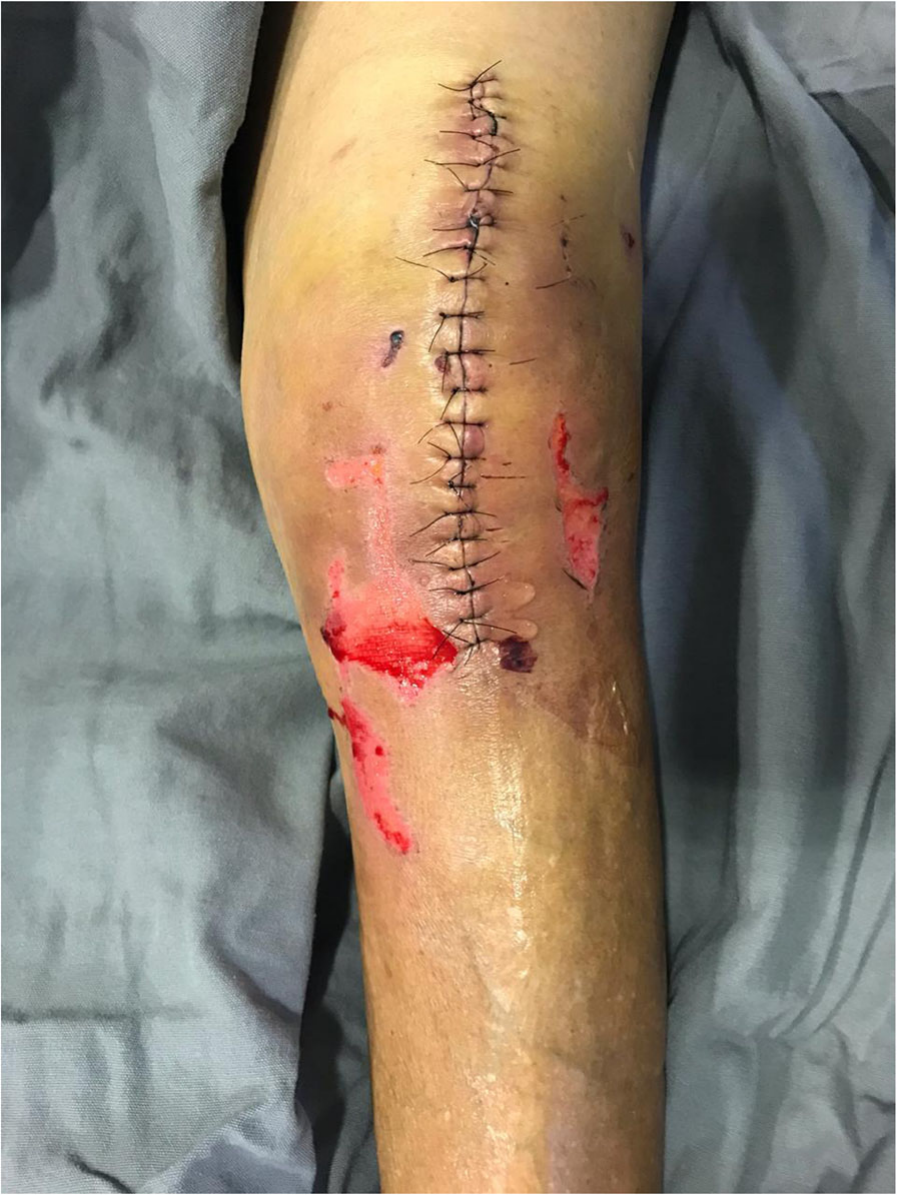
Blisters around the surgical wound of a patient with rheumatoid arthritis and diabetes who underwent total primary arthroplasty of the left knee and used conventional dressings; 14 days postoperatively

**Fig. 5:**
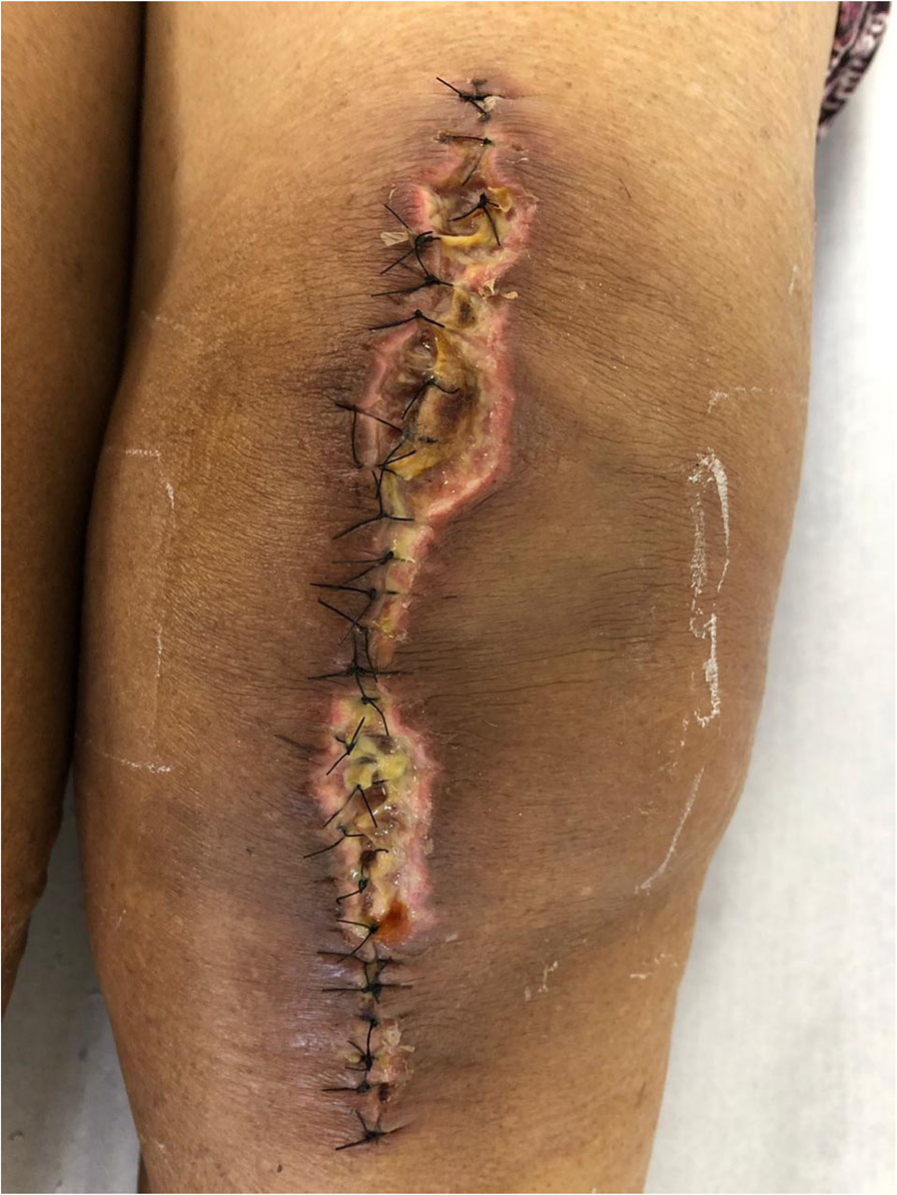
Dehiscence of the surgical wound associated with prosthesis infection in a patient with rheumatoid arthritis who underwent primary total arthroplasty of the left knee and used conventional dressings; 14 days postoperatively

In addition to epidemiological data and comorbidities, the following parameters related to the surgical wound were evaluated: presence of a haematoma around the wound or operated knee; persistent drainage from the surgical wound (considered as drainage for more than 7 days); hyperaemia around the surgical wound, either focal or widespread, defined as any redness around the skin incision, skin necrosis (Fig. 2), independent of requiring therapeutic treatment; dehiscence of the surgical wound (Fig. 3), superficial or deep, independent of requiring therapeutic treatment; presence of blisters around the surgical wound (Fig. 4); presence of postoperative infection (Fig. 5); length of hospital stay; and need for further intervention for any reason. Reintervention was considered as the need to take the patient to the operating room for any reason related to the arthroplasty. The incidence of deep venous thrombosis (DVT) was also recorded.

The presence or absence of all parameters related to the wound were performed by two evaluators dichotomously (yes or no). The more experienced examiner served as the main examiner, and the less experienced examiner enabled evaluation of interrater agreement.

### Data and statistical analyses

Separate univariate and multivariate analyses were performed for the full cohort of patients.

For the univariate analysis, Pearson’s chi-square test and the Fischer test for categorical variables were performed. For continuous variables, Shapiro-Wilk tests and histogram analysis were used to check the normality of the data, and the independent samples t test or Wilcoxon rank-sum test was used for continuous variables as appropriate.

We used multivariate analysis with logistic regression to assess the independent risk factors for the presence of any complication, including infection, wound dehiscence, wound edge necrosis, blisters or persistent wound drainage.

No a priori power calculation was performed, as all available eligible subjects were included. A post hoc power calculation revealed an achieved power of 91.2% for the comparison of any complication between all patients using NPWT and the control group. For the logistic regression, the practical rule that there should be at least 10 events per variable was considered, and therefore, the sample was adequate.

The interrater agreement between the 2 examiners for the wound parameters was evaluated using the Cohen kappa coefficient.

All normally distributed continuous variables are reported as the mean +/− standard deviation, and non-normally distributed variables are presented as the median (interquartile range). Statistical significance was considered when the *p* value was less than 0.05. The statistical software SPSS 22 (IBM Corp., NY, USA) and G*Power 3.1.9.3 (Erdfelder, Faul, & Buchner, Universität Düsseldorf, Düsseldorf, Germany 2009) were used to perform the analysis.

## Results

A total of 101 patients who underwent TKA in which NPWT was used were initially included, but 4 (3.9%) patients were later excluded because they could not perform the appropriate follow-up on the pre-established days. This group of 97 patients (Group 1) was compared with a historical control group of 199 patients who underwent the same surgical procedure but used conventional dressings postoperatively (Group 2). In group 2, the initial sample consisted of 208 patients, but 9 (4.3%) patients that did not accomplish the correct follow-up. The final sample for this study included 296 patients.

The epidemiological characteristics of the included patients are summarized in Table 1. The groups did not differ in regard to sex, age or clinical comorbidities. The occurrence of comorbidities was high in both groups: obesity, 22.7% vs 24.6% (*p* = 0.77); diabetes, 21.6% vs 15.1% (*p* = 0.19), smoking, 10.3% vs 6.0% (*p* = 0.23); and inflammatory diseases, 18.6% vs 16.6% (*p* = 0.39). A total of 153 (51.7%) patients had at least one risk factor for surgical wound complications, with 54 (55%) in group 1 and 99 (49%) in group 2, and 15 (15.5%) patients in group 1 and 20 (10.5%) patients in group 2 had at least 2 associated risk factors.

**Table 1:** Epidemiological characteristics of patients included in the study. BMI - body mass index. *RA* rheumatoid arthritis, *NPWT* negative-pressure wound therapy

	Total	NPWT	Conventional dressing	p
Male sex	37.2%	38.1%	36.7%	0.89
Age (years)	66.4	70 (15.5)	66 (13)	0.12
BMI	28.3	27.9 (2.7)	27.4 (4.5)	0.30
Obesity	24.0%	22.7%	24.6%	0.77
Diabetes	17.2%	21.6%	15.1%	0.19
Smoking	7.4%	10.3%	6.0%	0.23
RA	17.2%	18.6%	16.6%	0.39

The complications observed in each group are summarized in Table 2. The vast majority of adverse events that occurred in both groups were minor and had no major impact on the patients. The rate of infection in all patients was 2.4%, with 0% in the group that used NPWT and 3.5% in the group that used conventional dressings. Patients who used NPWT had a lower rate of total complications, minor or major (28.5% vs. 45.7%, *p* = 0.001), and less need to undergo a new intervention for any reason due to major complications in the operating room (2% vs. 8.5%, p = 0.001). The 2 reinterventions in group 1 occurred for aspiration of a haematoma in one case and for superficial cleaning of the surgical wound with placement of a new negative-pressure dressing in another case. In group 2, 17 reinterventions occurred: 5 cases of aspiration or drainage of a haematoma, 4 cases of interventions in non-infected surgical wounds with dehiscence, 7 cases of surgical cleaning with a polyethylene insert exchange due to infection and 1 case of washing and closing of a fistula at the surgical drain site.

**Table 2:** General complications. NPWT - negative-pressure wound therapy

	Total	NPWT	Conventional dressing	p
Haematoma	4.1%	2.1%	5.0%	0.34
Persistent drainage	4.1%	1.0%	5.5%	0.11
Hyperaemia	35.1%	24.7%	40.2%	0.01
Necrosis	6.4%	2.1%	8.5%	0.04
Dehiscence	7.8%	3.1%	10.1%	0.03
Blister	3.4%	1.0%	4.5%	0.17
Infection	2.4%	0.0%	3.5%	1
Any Complication	39.2%	28.5%	45.7%	0.001
Length of stay (days)	4.2	3 (1)	3 (1)	0.56
Reintervention	6.4%	2.0%	8.5%	0.001

**Fig. 6:**
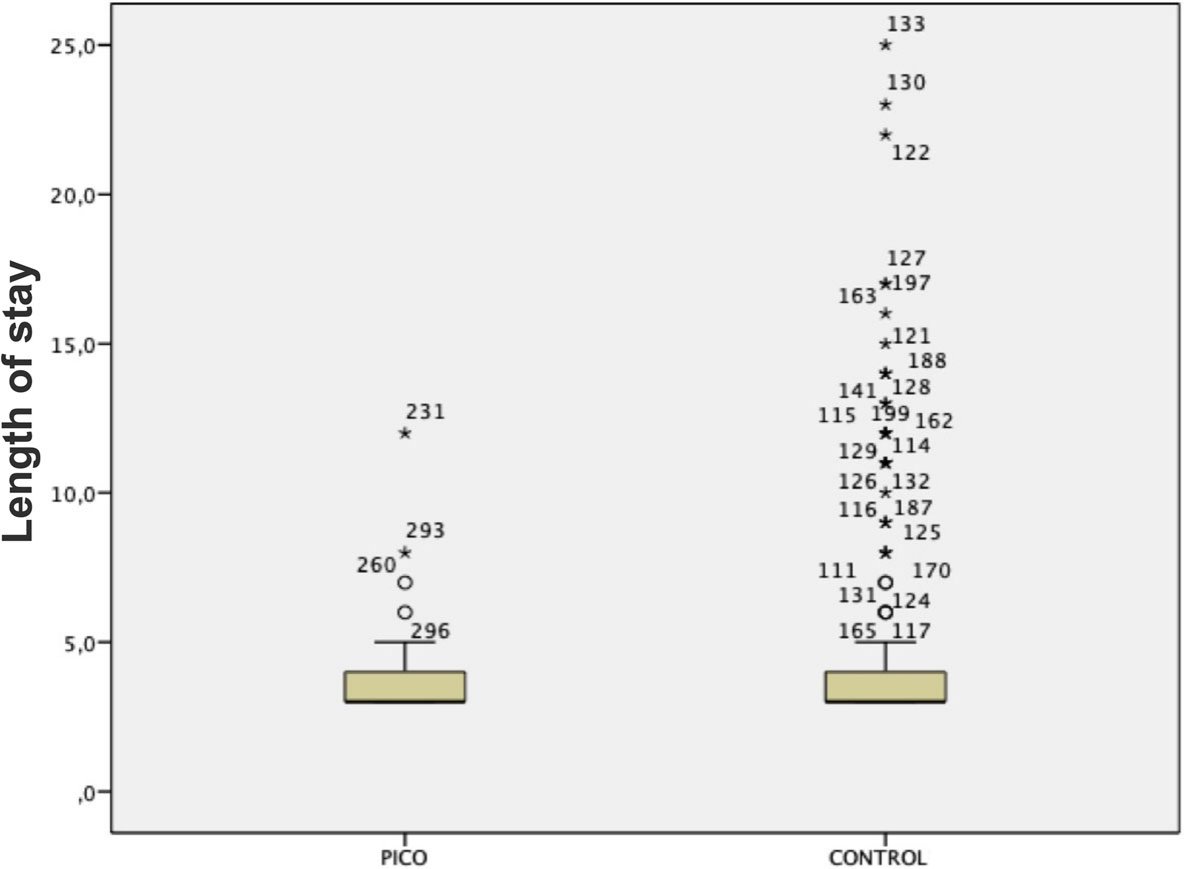
Length of hospital stay among the PICO and control groups. Although the median between groups was similar, the number of outliers in the control group was much higher, making the length of stay of patients who used conventional dressings more unpredictable

The most prevalent complication was hyperaemia around the surgical wound (35.1%). Patients who used NPWT had a lower incidence of hyperaemia (14.7% vs. 40.2%, *p* = 0.01), lower rate of necrosis (2.1% vs. 8.5%, *p* = 0.04) and lower rate of skin dehiscence (3.1% vs 10.1%, *p* = 0.03). The presence of haematoma, persistent drainage, blister and infection was not significantly different between the groups; however, these parameters had lower absolute numbers in the group that used NPWT. Length of hospital stay was not different between the groups, despite the high number of outliers in the control group (Fig. 6).

The correlation coefficient to assess inter-examiner agreement regarding the wound parameters evaluated varied from 0.74 to 0.92, and were considered from substantial to almost perfect agreement.

Logistic regression was used for the independent assessment of risk factors (diabetes, smoking, inflammatory arthritis and obesity) and of the use of NPWT in the presence of complications. Of these, the use of NPWT and inflammatory arthritis were statistically significant (*p* < 0.001 and *p* = 0.001). The use of NPWT was a protective factor for the presence of complications, with an odds ratio of 0.36 (95% CI 0.206–0.629).

The overall incidence of DVT was 3.4% and it was similar between the two studied groups (3.1% for Group 1 and 3.5% for Group 2).

## Discussion

The main findings of this study are the high rate of complications, most of which were minor, related to the surgical wound of patients undergoing TKA and the potential benefit of using NPWT to reduce these adverse events and the need for reintervention in these patients.

Most studies that evaluate complications in arthroplasty only report major complications, such as infection or deep dehiscence that require reopening of the surgical wound. Less severe complications are often underreported. Sharma et al. [21], in a systematic review that evaluated the best type of dressing for knee and hip arthroplasties, showed that only 5 of 12 studies evaluated hyperaemia around the wound, and only 1 of 12 studies evaluated haematoma; therefore, the actual occurrence of these minor wound complications is unknown. Despite this, Frosch et al. [22], in a study of 567 arthroplasties, showed a rate of major and minor complications of 23.6%, with the most frequent being delayed wound healing, but of these cases, only 5.6% required reintervention. Kim et al. [23], in a study with patients with central sensitization, also reported a higher rate (28.6%) of wound complications after arthroplasty.

In our study, despite the high number of reported complications, most were only hyperaemia around the surgical wound, a frequently under reported complication, which makes it difficult to compare our findings with current literature. A recent systematic review and meta-analysis by Kim et al. found an absolute risk of wound complications of 9.3% for patients that used NPWT and 12.8% for conventional dressings, although authors did not stratify each complication and probably did not consider minor complications [24]. On the other hand, Helito el al [25]., in a study that evaluated the effects of nasal oxygen catheter supplementation on wound healing after TKA, showed a rate of hyperaemia around the surgical wound of 54.1%. In this study, all cases that progressed to some major complication initially exhibited hyperaemia around the wound, and 34.4% of patients who initially presented hyperaemia progressed to another complication, hence the importance of avoiding hyperaemia around the surgical wound. Although hyperaemia around the wound most often does not represent a problem, it cannot be neglected because it is an early event that can progress to superficial wound infection according to the Centers for Disease Control (CDC) [26]. The use of negative-pressure therapy in our study was able to reduce the number of patients with this complication.

Another cause of the high number of complications is the incidence of patients with comorbidities that may increase the risk of delayed wound healing and infection after arthroplasty. Obese, diabetic patients, smokers and those with rheumatoid arthritis are known to have an increased risk of complications compared to patients without comorbidities [9–13]. The rate of these pathologies in our case series was greater than 50, and 11.8% of the cases had at least 2 comorbidities.

The use of NPWT effectively reduced the rate of complications. Although the infection rate was not significantly different between the groups, group 1 did not present any case that evolved with prosthesis infection. In addition, the total number of complications and parameters, such as hyperaemia, edge necrosis and suture dehiscence, occurred less frequently in group 1. Although the vast majority of these complications did not require reintervention, the reintervention rate in patients who used NPWT was lower, which certainly generates lower cost for the payer, although this was not evaluated in detail in this study. Recent cost-effectiveness studies have shown that the use of NPWT is cost-effective, especially in high-risk patients [27, 28]. Regarding length of hospital stay, although there was no significant difference between groups, group 2 had a large number of outliers, unlike group 1. Karlakki et al. [29] reported greater predictability in length of hospital stay for patients who received NPWT. Kim et al. also showed less reoperation rates and reduced length of stay in patients who used NPWT [24].

Similar studies have been conducted for the use of NPWT in the period after arthroplasty, with conflicting results. Redfern et al. [19] evaluated the rate of complications after primary knee and hip arthroplasties and showed a lower rate of complications, despite a similar incidence of infection, similar to that found in this study. Manoharan et al. [20], in turn, also in a study on primary knee arthroplasty, did not find any positive effects on surgical wound healing. In cases of revision arthroplasty, the results favour the use of NPWT, perhaps due to the increased risk of complications in this patient population. Cooper and Bas [30] showed a lower rate of wound complications and a lower rate of periprosthetic infection, and Newman et al. [31] showed a lower rate of complications in high-risk patients who used NPWT. Cooper et al. also reported a lower rate of complications in patients undergoing NPWT after periprosthetic fracture [32].

The main limitation of the present study is the fact that the operations and follow-up occurred on different dates. Even though the surgeries were performed by the same group of surgeons and with the same surgical technique, surgical skills, especially regarding soft tissue protection techniques, might be improved during this four-year period. Another limitation is that the groups presented different number of patients, nevertheless the number of patients was satisfactory for the proposed analysis. Although a sample size calculation was not performed initially, the post hoc power of the study was 91.2%, which is considered quite satisfactory.

## Conclusion

The number of complications related to the wound after TKA is high; however, most of them are minor and have no impact on the treatment and clinical evolution of patients. The use of NPWT decreased the number of surgical wound complications, especially hyperaemia, dehiscence and necrosis, and reduced the need for reintervention.

## Data Availability

The datasets generated and/or analyzed during the current study are not publicly available because it belongs to the personal data of our institution and are used in other articles related to this topic but are available from the corresponding author on reasonable request.

## Abbreviations

TKA: Total knee arthroplasty
NPWT: Negative-pressure wound therapy
PCL: Posterior cruciate ligament
DVT: Deep venous thrombosis
CDC: Centers for Disease Control
